# Simulation Study of High Sensitivity Fiber SPR Temperature Sensor with Liquid Filling

**DOI:** 10.3390/s22155713

**Published:** 2022-07-30

**Authors:** Min Xiong, Chuanxin Teng, Ming Chen, Yu Cheng, Shijie Deng, Fuwang Li, Hongchang Deng, Houquan Liu, Libo Yuan

**Affiliations:** 1 Guangxi Key Laboratory of Optoelectronic Information Processing, Guilin University of Electronic Technology, Guilin 541004, China; xiongmin0301@163.com (M.X.); xinchuanteng@126.com (C.T.); mchenqq2011@163.com (M.C.); shijie.deng@guet.edu.cn (S.D.); li15650577993@163.com (F.L.); hcdeng@guet.edu.cn (H.D.); liuhouq@guet.edu.cn (H.L.); lbyuan@vip.sina.com (L.Y.); 2 College of Photonic and Electronic Engineering, Guilin University of Electronic Technology, Guilin 541004, China

**Keywords:** fiber SPR, thermo-optic coefficient, surface plasmon

## Abstract

In this paper, a high sensitivity fiber temperature sensor based on surface plasmon resonance is designed and studied. In the simulation, the single mode fiber is polished to remove most of the cladding, and then gold and silver films are added. Finally, it is embedded in the heat shrinkable tube filled with a thermo-optic coefficient liquid for curing. The numerical simulation results show that the sensing characteristics are sensitive to the remaining cladding thickness of the fiber, the thickness of the gold film and the thickness of the silver film. When the thermo-optic coefficient of the filling liquid is −2.8 × 10^−4^/°C, the thickness of the gold film, the thickness of the silver film and the thickness of the remaining cladding of the fiber are 30 nm, 20 nm and 1 μm, respectively. The sensitivity of the sensor designed in this paper can reach −6 nm/°C; this result is slightly higher than that of similar research in recent years. It will have a promising application prospect in flexible wearable temperature sensors, smart cities and other fields.

## 1. Introduction

Fiber temperature sensing has good biocompatibility and real-time data acquisition, which can fully meet the needs of a complex environment and testing requirements [1,2,3,4,5]. At present, a variety of fiber temperature sensors have been studied, such as fiber grating temperature sensors [3,6,7], Mach–Zder interference (MZI) sensors [8,9], and Fabry–Perot interferometer (FPI) fiber temperature sensors [10,11], etc. These studies have extensively promoted the development of temperature sensors. However, some problems still exist, including the sensitivity and manufacturing cost not being satisfactory. In recent years, researchers have found that fiber temperature sensors based on surface plasmon resonance (SPR) have a higher sensitivity than traditional ones [12]. Therefore, many related kinds of research have been explored continuously. Photonic crystal fibers have been extensively studied due to their diverse structural designs [13,14,15,16,17,18,19]. For instance, in 2018, Wang Yong et al. [17] proposed a temperature sensor composed of gold-plated multimode fiber-photonic crystal fiber multimode fibers (MMF-PCF-MMF), and a sensitivity of −1.551 nm/°C was achieved in the temperature range of 35–100 °C by using PDMS coating. In addition, some fiber optic SPR sensors filled with special liquids have also been studied. Chen Ao [13] reported a temperature sensor based on D-shaped photonic crystal fibers (PCF). A gold film was plated on an air hole near the center of the fiber core, and liquid ethanol with a thermal conductivity was filled to detect temperature. The results showed that the sensitivity reached 1.075 nm/°C in the range of 20–60 °C. At the same time, an alcohol-filled optical fiber based on hollow fiber was proposed [15], and its linear sensitivity in the range of 35–70.1 °C was as high as 1.16 nm/°C. A high-sensitivity D-type double-clad optical fiber temperature sensor based on surface plasmon resonance [20], in which the D-type cross-section was plated with a gold film, and the liquid with a high thermal coefficient was injected, had realized a temperature sensitivity of 3.635 nm/°C. In addition, a SPR thermometer based on liquid crystal (LC) filled hollow fiber has also been demonstrated [21]. In recent years, researchers have been trying to study how to improve the sensitivity of temperature sensors and expand the detection temperature range.

It is well known that gold and silver are the two most suitable materials to excite SPR due to their unique dispersive properties. In this paper, a SPR temperature sensing probe sealed in a capillary filled with a thermosensitive solution is proposed. The simulation involves mechanically throwing away most of the cladding and coating with gold and silver mixed metals. When the external ambient temperature changes, according to the thermo-optic coefficient of the filled thermosensitive solution, the refractive index of the liquid inside the capillary also changes, resulting in a shift in the resonance wavelength. Therefore, we can establish a functional relationship between the SPR resonance wavelength and temperature, and thus realize temperature sensing. Through the finite element simulation, the effects of the remaining cladding thickness of the fiber, the thickness of the gold film and the thickness of the silver film on the temperature sensing characteristics are studied.

## 2. Structural Design and Optimization

**Figure 1:**
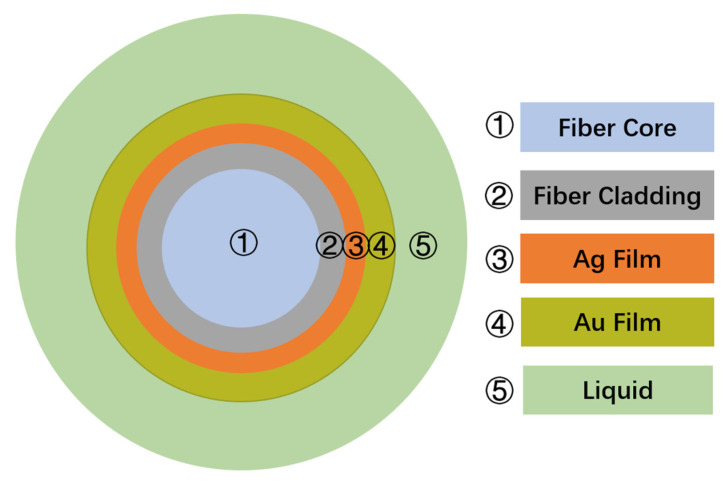
Cross section of the optical fiber temperature sensor.

The structure of fiber temperature sensor is shown in Figure 1, mainly composed of a single-mode optical fiber, metal films, and liquid filled with thermo-optic coefficient. In the simulation, after mechanically throwing away the cladding, the remaining thickness of fiber cladding is described by *d_cladding*, where the core diameter of the optical fiber is *d_core* = 10 μm, the core refractive index is 1.4681, and the cladding refractive index is 1.4628. The thicknesses of gold and silver are *d_Au* and *d_Ag*, respectively. According to the Drude dispersion model of the metal, the expressions are as follows [22]:

(1) $$\varepsilon (\lambda )=1-\frac{\lambda^{2}\lambda_{c}}{{\lambda_{p}}^{2}(\lambda_{c}+i\lambda )}$$

In the above formula, $\lambda_{c}$ represents the collision wavelength, $\lambda_{p}$ is the wavelength corresponding to the plasma frequency, and λ represents the wavelength of incident light.

The values corresponding to $\lambda_{c}$ and $\lambda_{p}$ are shown in the following Table 1:

The thermo-optic coefficient and expansion coefficient of silica are 7.8 × 10^−6^/°C and 4.1 × 10^−7^/°C, respectively [23]. Assuming the thermo-optic coefficient of the liquid as dn/dT, the relationship between the refractive index of the filled liquid and the temperature can be evaluated by [24]

(2) $$n_{\det}=n_{\det0}+(T-T_{0})dn/dT$$

where the $n_{\det0}$ is the refractive index of the filling liquid at room temperature *T*_0_. It can be obtained from the literature that when the ratio of ethanol and glycerol are 34% and 66%, respectively, and when the temperature is 25 °C, the refractive index is 1.436 and the thermo-optic coefficient is −2.8 × 10^−4^/°C [20,25].

In the fiber SPR temperature sensor designed in this paper, a broad-spectrum light source is injected into the optical fiber. When the light is experiencing total internal reflection in the sensing area, the evanescent field on the surface of the metal film can excite surface plasmon waves, which attenuate at specific wavelengths, so that the resonance absorption peak can be observed in the transmission curve. When the ambient temperature changes, the refractive index of the liquid encapsulated inside the optical fiber changes accordingly due to the thermo-optical coefficient, which leads to a shift of the resonance wavelength, and then the functional relationship between SPR resonance wavelength and temperature can be established. Here, the transmittance is calculated as [26]

(3) $$T(\lambda )=\exp(-\frac{4\pi }{\lambda }Im(n_{eff})L)$$

where $n_{eff}$ represents the effective refractive index, $I_{m}$ represents its imaginary part, and *L* is the length of the sensing area; this paper takes *L* = 0.1 mm.

As we all know, the most important parameters to evaluate the performance of a sensor are sensitivity and half-peak width. Here, the sensitivity formula is as follows [20]:

(4) $$S_{\lambda }=\frac{d\lambda_{\mathrm{res}}(T)}{dT}[\mathrm{nm}/{}^{\circ}C]$$

However, the simulation results show that at long wavelengths, due to the broadening of the transmission spectrum curve, the wavelengths corresponding to the left and right sides in the half-peak width formula cannot be obtained. Therefore, we discard the half-peak width and choose the transmittance corresponding to the resonance peak as another evaluation standard. 

## 3. Results and Discussion

**Figure 2:**
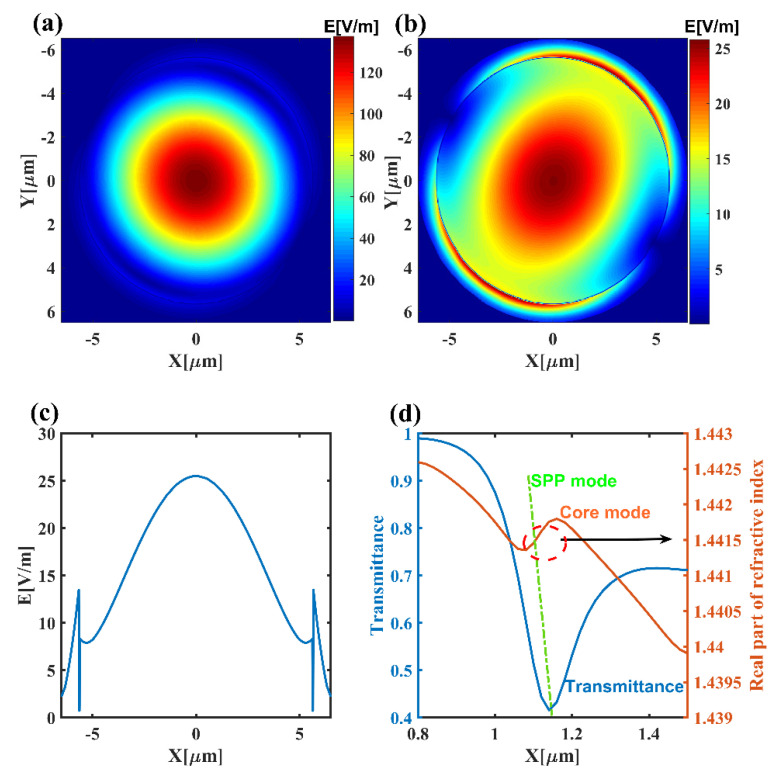
(**a**) Electric field diagram of the fundamental mode of fiber at 800 nm. (**b**) Electric field diagram of excited SPP. (**c**) Electric field enhancement diagram of fiber cross-section at SPR. (**d**) Real part of effective refractive index of fiber core mode and SPP mode, and transmission of fiber SPR sensor.

Figure 2 shows the electric field distribution of the fundamental mode and the SPP electric field, the effective refractive index of the core mode and the surface plasmon polaritons (SPP) mode, and the transmission of the fiber SPR sensor, when *d_cladding* = 1 μm, *d_Ag* = 30 nm, *d_Au* = 10 nm, and the external environment is 50 °C. It can be seen in Figure 2a that the optical field is mainly concentrated in the core area, and no SPR phenomenon occurs. However, when the wavelength is 1150 nm, as shown in Figure 2b, the evanescent field is enhanced at the interface between the metal and the external environment; when the SPR effect occurs, the energy at the symmetrical sides of the cross section is locally enhanced, but not at the circular interface of the whole fiber. This is because only P-polarized light can produce SPR, while S-polarized light does not produce SPR [26]. As can be seen from Figure 2c, the light field decays rapidly inside the metal, but at the interface between the metal and the external environment, the electric field strength rebounds and increases sharply, which indicates that the SPR phenomenon appears. Figure 2d shows the effective refractive index *Re(n_eff_)* of the fiber core mode and the effective refractive index *Re(n_spp_)* of the SPP mode, and the transmittance of the fiber SPR temperature sensor at different wavelengths. As shown in the Figure 2d, the green dashed line for *Re(n_spp_)* shows a linear drop, while the red solid line for *Re(n_eff_)* exhibits an S-shaped kink. For circularly polished fibers, as the wavelength increases, the electromagnetic field is revealed more into the cladding, which leads to a decrease in *Re(n_eff_)*. However, with the appearance of plasma resonance, the core mode has changed due to evanescent fields and resonance electron oscillations. The electromagnetic field on the left side of the resonance wavelength is called the slow electromagnetic field, which is accelerated by resonance. On the contrary, the electromagnetic field on the right side of the resonant wavelength is called the fast electromagnetic field, which is slowed down by resonance. Because of this, it can be judged that the resonant wavelength appears in the middle of the S-shaped kink of *Re(n_eff_)* [27]. Meanwhile, when the wavelength equals 1150 nm, *Re(n_eff_)* = *Re(n_spp_)*. At the same time, the transmission peak reaches the minimum, as shown by the solid blue line, so it can be judged that the resonance wavelength is 1150 nm.

**Figure 3:**
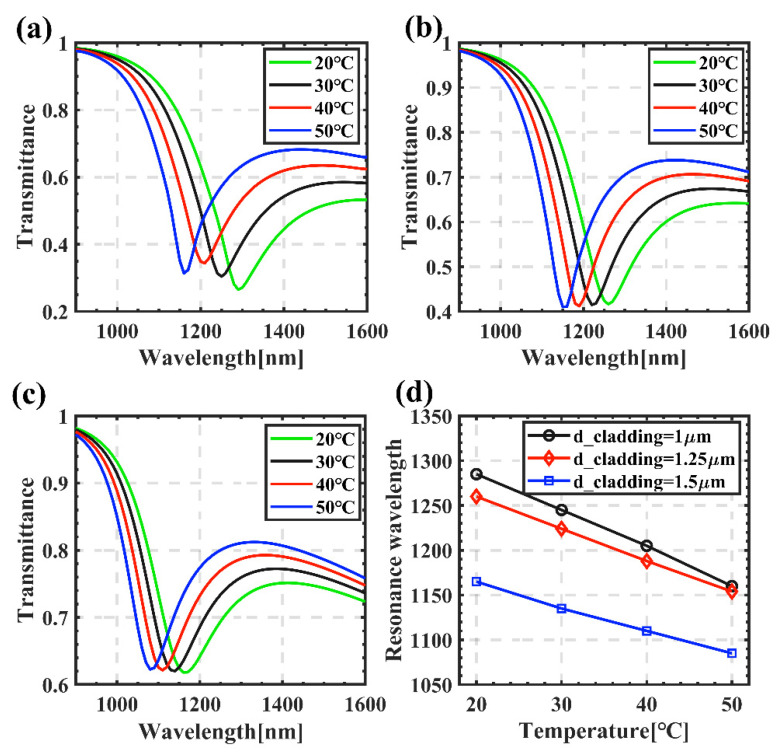
Influence of *d_cladding* on the transmittance spectra: (**a**) *d_cladding* = 1 μm; (**b**) *d_cladding* = 1.25 μm; (**c**) *d_cladding* = 1.5 μm. (**d**) The fitted results resonant wavelength with different *d_cladding*, where the black, red and blue markers are the simulation results.

Since the gold–silver mixed film is selected as the metal layer in this paper, and the fiber SPR are mainly affected by the type of metal film, the thickness of the metal and the thickness of the cladding, we specifically discuss and analyze the above variables. The *d_cladding* affects the strength of the evanescent field and thus affects the sensing characteristics. Figure 3 shows that the resonance wavelength gradually blue-shifts with increasing temperature, corresponding to different *d_claddings*. When *d_cladding* = 1 μm, as the temperature increases from 20 °C to 50 °C, the resonance wavelength moves from 1360 nm to 1150 nm, and the transmittance corresponding to the resonance peak gradually increases. For example, when *d_cladding* = 1 μm, 1.25 μm and 1.5 μm, respectively, the corresponding resonance peak transmittance at 20° rises from approximately 0.24 to 0.63. This is mainly because with the increasing of *d_cladding*, the energy of the light field is more confined in the core, and the evanescent field decreases, which weakens the plasma wave [20]. Figure 3d shows the fitting curves of the resonance wavelengths under different *d_claddings*. It can be seen that the offset of the resonance wavelengths has a good linear relationship with the temperature. When *d_cladding* is 1 μm, 1.25 μm and 1.5 μm, its sensitivity reaches −6 nm/°C, −3.6 nm/°C and −2.8 nm/°C, respectively. At the same time, because the cladding binds the light in the core for transmission, too thin cladding is not conducive to transmitting light, and it is easy to damage the fiber core during the preparation process. Therefore, considering the temperature sensitivity and the transmittance of the resonance wavelength, we choose *d_cladding* = 1 μm as the basis for further study.

**Figure 4:**
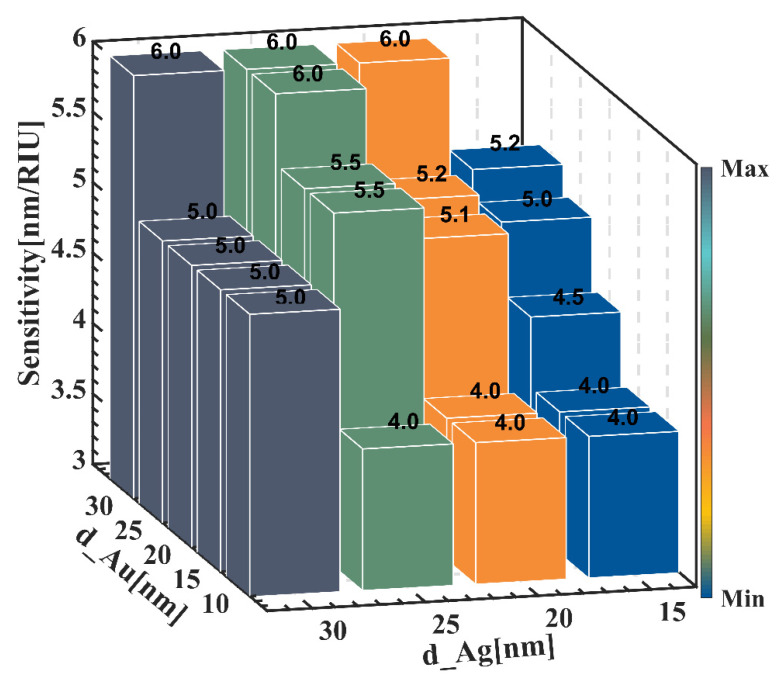
Sensitivity corresponding to different gold and silver film thicknesses.

To discuss the effect of the thickness of metal on the sensing characteristics, by changing *d_Au* from 10 nm to 30 nm with a pitch of 5 nm, and *d_Ag* from 15 nm to 30 nm with a pitch of 5 nm, the sensing curves for a total of 20 cases are simulated. A promising sensor usually requires higher sensitivity as well as lower transmittance. Higher sensitivity indicates the accuracy of detection of sensing characteristics, and lower transmittance means that the detection signal is more distinguishable and less susceptible to interference from extraneous signals. As can be seen from Figure 4, when *d_Ag* is fixed, the overall sensitivity increases slightly with increasing *d_Au*. For example, when *d_Ag* = 15 nm, the corresponding maximum sensitivity increases from 4 nm/°C to 5.2 nm/°C when *d_Au* grows from 10 nm to 30 nm, an improvement of nearly 160%; while at other thicknesses, such as *d_Ag* = 20 nm, the total sensitivity also increases by 150% with the increase of *d_Au*. At the same time, we found that when the total metal film thickness was larger, the sensitivity was significantly higher than when the metal film thickness was lower. This is mainly because the increase of total metal film thickness prolongs the light propagation distance in the structure, thus generating more surface equipartition excitations, which in turn enhances the electric field strength between the dielectric surface and the external environment and improves the sensitivity [28].

**Figure 5:**
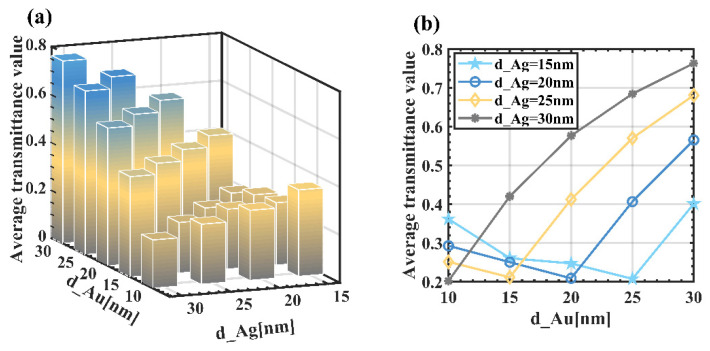
Average value of minimum transmittance under different metal layers.

Therefore, to obtain high temperature sensitivity, the total thickness of the metal film should be between 40–50 nm, and *d_Au* should be greater than 20 nm. However, high sensitivity does not mean that the sensor has excellent performance. Figure 5 shows the average transmittance of the resonant wavelength under different film thicknesses. It can be seen from Figure 5a that when the total film thickness is less than 40 nm, the average transmittance value remains small. However, when the total metal film thickness exceeds 50 nm, the average value of transmission will be greater than 0.50. As shown in Figure 5b, it can be seen that with the increase of *d_Au*, the average transmission decreases first and then increases. For example, when *d_Ag* = 15 nm is fixed, and when *d_Au* increases from 10 nm to 30 nm, the transmittance firstly decreases from 0.36 to 0.20. When d_Au exceeds 25 nm, that is, when the total thickness of the metal film exceeds 40 nm, the transmittance quickly rebounds up to 0.40, increasing by nearly 200%.

For several other cases, the trend is the similar, as shown in Figure 5a; for example, when *d_Au = d_Ag* = 30 nm, the average transmittance of its resonance wavelength reaches 0.76, which is extremely unfavorable to the differentiation of resonance peak wavelength. The trend of transmittance can be attributed to the fact that as the thickness of the metal film increases, the evanescent field increases in the metal film. When the metal film thickness is too thick, it also exceeds the penetration depth of the evanescent field, resulting in a lower coupling of the surface plasma wave (SPW) and evanescent field energy, which leads to a lower intensity of the SPR peak. Therefore, combining the sensitivity and transmittance, we chose the total thickness of the gold and silver film equal to 40 nm as the main basis of the study.

**Figure 6:**
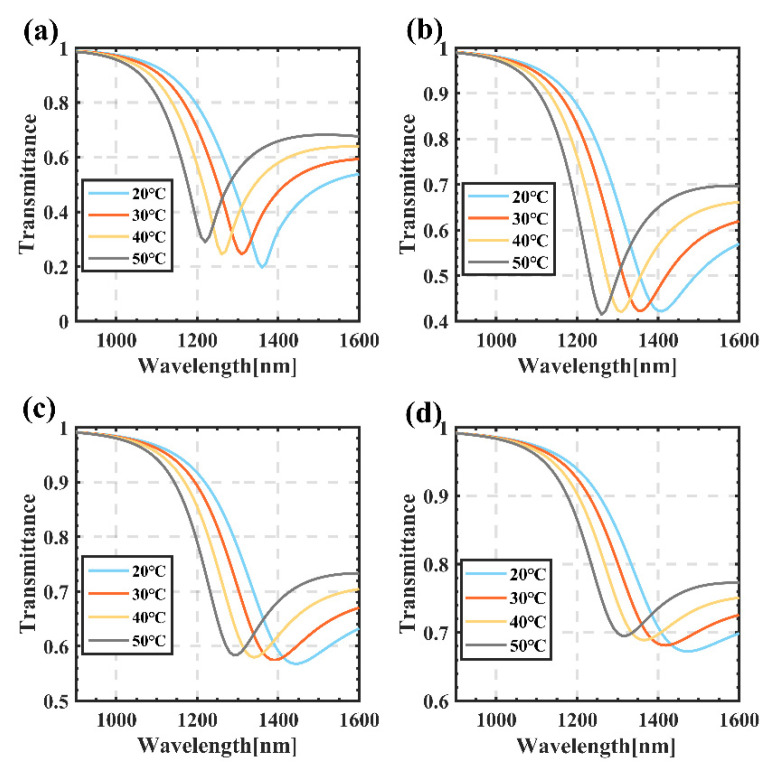
Influence of *d_Ag* and *d_Au* on the transmittance spectra: (**a**) *d_Ag* = 30 nm, *d_Au* = 10 nm; (**b**) *d_Ag* = 30 nm, *d_Au* = 15 nm; (**c**) *d_Ag* = 30 nm, *d_Au* = 20 nm; (**d**) *d_Ag* = 30 nm, *d_Au* = 25 nm.

In order to describe the effect of different *d_Au* on the sensing characteristics, we chose four groups of sensing curves when *d_Ag* = 30 nm, *d_Au* = 10 nm, 15 nm, 20 nm and 25 nm, as shown in Figure 6. With the increase of temperature, the resonance wavelengths of the spectra corresponding to the four groups of parameters show a blue shift, but their resonance wavelengths are not the same; e.g., when *d_Ag* = 30 nm and *d_Au* = 10 nm, their resonance wavelengths are mainly concentrated at 1220~1360 nm. Conversely, for the other groups, as shown in Figure 6d, corresponding to *d_Ag* = 30 nm and *d_Au =* 25 nm, their resonance wavelengths are mainly located at 1320–1470 nm. This is mainly due to the fact that the dielectric constant of the mixed metal affects the resonance wavelength, while it can be seen that the minimum transmittance gradually increases with the increase of the total thickness. The minimum transmittance in Figure 6a is mainly distributed around 0.20, while in Figure 6d, it is around 0.75, and the spectrum of the transmittance is gradually broadened; as such, the half-peak width cannot be obtained, which is the main reason why the half-peak width is not selected in this paper. 

To assess the fiber optic SPR temperature sensing characteristics of our design, Table 2 shows a comparison between the proposed work and state of the art. There has been a lot of research work on fiber optic temperature sensing in the last decade, and here we just list some temperature sensors based on the fiber SPR simulation. We analyze the year of publication and structure, as well as the temperature range and sensitivity. From the data in the table, we can see that the designed sensor has slightly improved in sensing sensitivity compared to other studies. However, due to the limitation of the thermo-optical coefficient of the ethanol and glycerol mixture used in this paper, our measurement range is currently lower than some of these studies. In the future, liquid-filled materials will be investigated to pursue a wider temperature measurement range and higher sensitivity.

## 4. Conclusions

In conclusion, a new type of high sensitivity fiber SPR temperature sensor was designed and analyzed in this study. The resonant wavelength of the sensor will change correspondingly due to the thermo-optic coefficient of the filling liquid. The thicknesses of the metal layer and cladding are discussed by finite element simulation, and finally a fiber SPR sensor with a temperature sensitivity of −6 nm/°C in the range of 20–50 °C is proposed. The sensitivity is slightly improved compared to existing studies, and the research will provide technical support for temperature measurements in complex environments.

## Figures and Tables

**Table 1:** Material properties of gold and silver.

Dispersion Coefficients (μm)	Metal	
	Ag	Au
$\lambda_{c}$	0.14541	0.16826
$\lambda_{p}$	17.614	8.9342

**Table 2:** Comparison table of performance parameters.

Reference	Year	Structure	Temperature Range (℃)	Sensitivity (nm/℃)
Ref. [14]	2016	PCF and Au	0–100	−3.08
Ref. [20]	2017	SMF and Au	20–50	−3.635
Ref. [15]	2020	HCF and Ag	35.5–70.1	−1.16
Ref. [29]	2021	SMF and Au	35–95	−1.765
Ref. [30]	2021	PCF and Ag	−15–35	−4.5
Ref. [31]	2021	SMF and Au	20–60	−2.41
Ref. [32]	2022	MF and Ag	20–60	−1.96
Our work	-	SMF and Au and Ag	20–50	−6.0
